# Lipidomics of the Edible Brown Alga Wakame (*Undaria pinnatifida*) by Liquid Chromatography Coupled to Electrospray Ionization and Tandem Mass Spectrometry

**DOI:** 10.3390/molecules26154480

**Published:** 2021-07-24

**Authors:** Davide Coniglio, Mariachiara Bianco, Giovanni Ventura, Cosima D. Calvano, Ilario Losito, Tommaso R. I. Cataldi

**Affiliations:** 1 Dipartimento di Chimica, Università degli Studi di Bari Aldo Moro, Via Orabona 4, 70126 Bari, Italy; davide.coniglio@uniba.it (D.C.); mariachiara.bianco@uniba.it (M.B.); giovanni.ventura@uniba.it (G.V.); ilario.losito@uniba.it (I.L.); 2 Centro Interdipartimentale SMART, Università degli Studi di Bari Aldo Moro, Via Orabona 4, 70126 Bari, Italy; 3 Dipartimento di Farmacia-Scienze del Farmaco, Università degli Studi di Bari Aldo Moro, Via Orabona 4, 70126 Bari, Italy

**Keywords:** seaweeds, wakame, algae, phospholipids, glycolipids, liquid chromatography, mass spectrometry

## Abstract

The lipidome of a brown seaweed commonly known as wakame (*Undaria pinnatifida*), which is grown and consumed around the world, including Western countries, as a healthy nutraceutical food or supplement, was here extensively examined. The study was focused on the characterization of phospholipids (PL) and glycolipids (GL) by liquid chromatography (LC), either hydrophilic interaction LC (HILIC) or reversed-phase LC (RPLC), coupled to electrospray ionization (ESI) and mass spectrometry (MS), operated both in high and in low-resolution mode. Through the acquisition of single (MS) and tandem (MS/MS) mass spectra more than 200 PL and GL of *U. pinnatifida* extracts were characterized in terms of lipid class, fatty acyl (FA) chain composition (length and number of unsaturations), and regiochemistry, namely 16 SQDG, 6 SQMG, 12 DGDG, 5 DGMG, 29 PG, 8 LPG, 19 PI, 14 PA, 19 PE, 8 PE, 38 PC, and 27 LPC. The FA (C16:0) was the most abundant saturated acyl chain, whereas the monounsaturated C18:1 and the polyunsaturated C18:2 and C20:4 chains were the prevailing ones. Odd-numbered acyl chains, iJ., C15:0, C17:0, C19:0, and C19:1, were also recognized. While SQDG exhibited the longest and most unsaturated acyl chains, C18:1, C18:2, and C18:3, in the *sn-1* position of glycerol, they were preferentially located in the *sn-2* position in the case of PL. The developed analytical approach might pave the way to extend lipidomic investigations also for other edible marine algae, thus emphasizing their potential role as a source of bioactive lipids.

## 1. Introduction

*Undaria pinnatifida* is a pluricellular life-form belonging to the group of *Algae*, composed of more than 30,000 aquatic, oxygen-evolving and photosynthetic autotroph organisms. *U. pinnatifida* is a brown seaweed [1], recognized as sea mustard and identified by its Japanese name *wakame*, which represents one of the most consumed edible algae cultivated in Japan since the Nara period (ca. 700 B.C.) [2]. Currently, it is extensively spread out thanks to the remarkable nutritional properties and epidemiological studies showing health benefits associated with seaweed consumption [3,4]. Wakame seaweed, like most algae, is rich in minerals as calcium, sodium, potassium, iron, magnesium, and iodine, vitamins, including vitamin B_12_, A, C, and E, fibers, and high-value proteins. Moreover, it represents an excellent source of phospholipids (PL) including fatty acyl chains featuring a valuable ω-3/ω-6 ratio. It is also characterized by a remarkable content of extra nutritional compounds, such as polyphenols, carotenoids, and fucoidans. Thanks to its worth bioactive compounds, wakame seaweed is not only employed as a foodstuff and seasoning but has been recently introduced as a supplement to daily diet [5,6,7,8,9,10] and for production of agar, carrageenan, and alginate [11]. Due to its extensive use, the nutritional properties, along with the occurrence of alkaloid [12] and phenolic compounds [13], the iodine and bromine contents [14], and safety concerns related to arsenic species [15] have been investigated.

**Figure 1:**
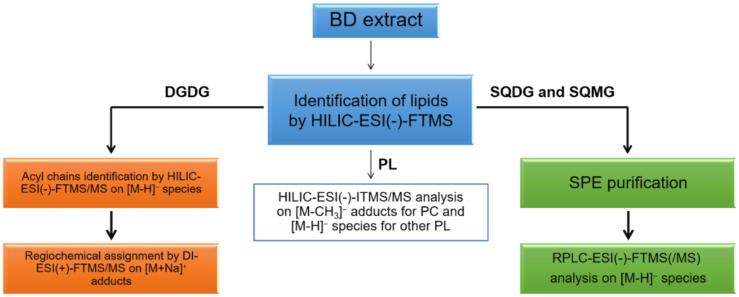
Adopted workflow: lipids from algal samples were extracted by using the procedure described by Bligh and Dyer. HILIC-ESI-(-)-FTMS(/MS) and HILIC-ESI-(-)-ITMS/MS were employed, in synergy, to characterize PL and DGDG; the regiochemistry of DGDG was assessed using direct infusion (DI)-ESI(+)-FTMS/MS analyses on the corresponding sodium adducts. RPLC-ESI(-)-FTMS/MS, following SPE-based purification, was adopted for the characterization of sulfur-containing glycolipids SQDG and SQMG.

Concerning lipid fraction, several studies on brown algae have been focused on minor arsenosugar phospholipids [16,17,18], arsenic-including hydrocarbons [19,20], and free polyunsaturated fatty acids [21,22]. While the knowledge of glycolipids (GL) and PL is foremost in red algae [23], these compounds have been only partially investigated in brown algae [24]. There has been extensive research regarding the fatty-acidome of algae; for instance, Řezanka et al. [25] focused on the characterization of total long-chain fatty acids in green algae *Botryococcus* by GC-MS. The most abundant FA were identified as three different kinds of C28 (i.e., 28:2, 28:1, and 28:0) and TAG, DAG, PA, and PC, containing these fatty acyl chains, were investigated by LC-ESI-MS/MS. As far as red algae, Peralta-García et al. [26] addressed the lipid profile of *Rhodymenia pseudopalmata* through thin layer chromatography, NMR, and GC-MS. Phospholipids were found to be the leading class in all the examined samples, while oleic and palmitic acids resulted as the main monounsaturated and saturated fatty acid, respectively. To achieve a more comprehensive characterization of their lipidome, two different chromatographic techniques, namely hydrophilic interaction liquid chromatography (HILIC) and reversed phase (RP)LC, both coupled to mass spectrometry (MS) by a heated electrospray (ESI) source, were employed in our laboratory to characterize GL and PL in the Bligh & Dyer extracts of *U. pinnatifida*. Evidence for the presence of phospholipids as phosphatidic acids (PA), phosphatidylethanolamines (PE), phosphatidylcholines (PC), phosphatidylglycerols (PG), and phosphatidylinositols (PI), and glycolipids as digalactosyldiacylglycerols (DGDG) and sulfoquinovosyl diacylglycerols (SQDG), both with their corresponding lyso-forms, were obtained during the present investigation. As emphasized in the workflow depicted in Figure 1, major information for PL assignment was recovered from HILIC separation followed by negative ion mode MS detection, whereas RPLC was very useful to investigate sulfoquinovosyl di- and mono-acylglycerols (SQDG, SQMG) and to distinguish the regioisomers of the latter that were scarcely separated by the HILIC column. Following earlier indications reported in the literature [27], direct infusion analysis in positive ion mode was carried out to assign the regiochemistry of DGDG. In the present paper, the lipidome characterization of *U. pinnatifida* seaweed based on the workflow reported in Figure 1 will be described in detail, giving special emphasis to the synergy between high- and low-resolution MS and MS/MS measurements.

## 2. Results and Discussion

### 2.1. Lipidomics of U. pinnatifida by HILIC-ESI(-)-FTMS

**Figure 2:**
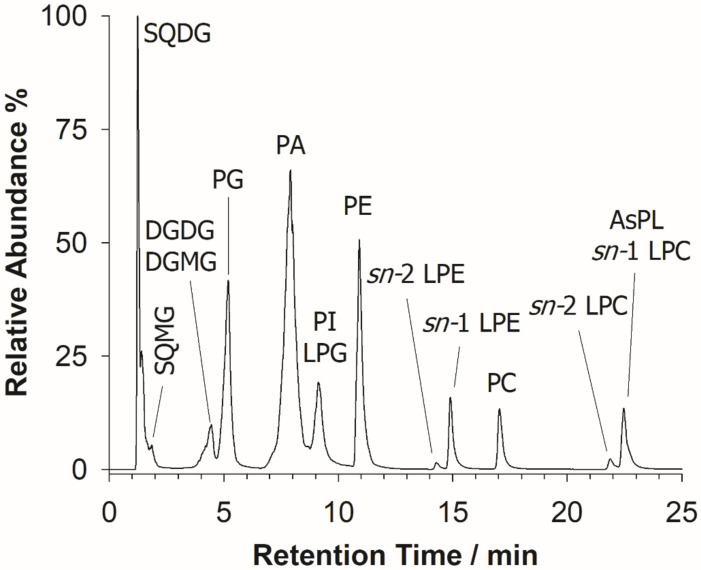
A typical total ion current (TIC) chromatogram arising from the HILIC-ESI(-)-FTMS full scan analysis of the BD extract of wakame seaweed. Sulfoquinovosyl diacylglycerols (SQDG), sulfoquinovosyl monoacylglycerols (SQMG), digalactosyl diacylglycerols (DGDG), digalactosyl monoacylglycerols (DGMG), phosphatidic acids (PA), phosphatidylinositols (PI), phosphatidylglycerols (PG), lyso-PG (LPG), phosphatidylethanolamines (PE), lyso-PE (LPE), phosphatidylcholines (PC), lyso-PC (LPC), and arsenosugar PL (AsPL) are labeled in the figure.

The characterization of the *U. pinnatifida* lipidome was initially performed by HILIC-ESI-FTMS, which provided total ion current (TIC) chromatograms like the one shown in Figure 2.

As expected, a separation based on the lipid class was typically observed [28]. The identification of lipids was accomplished using accurate *m*/*z* values, retrieved upon averaging the mass spectrum of each chromatographic peak/band, as input values for searches based on the Online Lipid Calculator (OLC, www.mslipidomics.info/lipid-calc/ accessed on 21 April 2021) or the Lipid Maps (www.lipidmaps.org/ accessed on 21 April 2021) databases, setting a mass accuracy ≤5 ppm. An output reporting the possible lipid class, along with the total number of carbon atoms and unsaturations (expressed with the C:U notation) of the fatty acyl chains included in each compound, was obtained. A lipid class confirmation was subsequently achieved for each lipid by the generation of specific class-related product ions in the corresponding tandem mass spectrum. The following lipid classes, eluting according to their relative chemical polarity, as expected for a HILIC column, were recognized: SQDG, sulfoquinovosyl monoacylglycerols (SQMG), DGDG, along with digalactosyl monoacylglycerols (DGMG), PA, phosphatidylglycerols (PG), PI, co-eluting with lyso-PG (LPG), PE, lyso-PE (LPE), PC, and lyso-PC (LPC). The most abundant PL and GL are summarized in Table 1. It is also worth noting that diacylarsenosugar phospholipids (As-PL), present at low concentrations and co-eluting with LPC, were also detected; their characterization has been recently described elsewhere [18].

To account for the relative abundance (r.a.) of each lipid species in a specific class, the related peak area was evaluated from the corresponding extracted ion current (XIC) chromatogram, then the sum of all peak areas for compounds detected in the class was calculated. It is important to point out that, although the HILIC run successfully separates lipid classes according to their polar head, it does not provide a good separation among lipids belonging to the same class [29,30]. SQMG, which are often present as couples of regioisomers differing for the acyl chain position on the glycerol backbone, were eluted rather close to the column dead volume. In this scenario, it was not possible to assign each isomer in a couple of regioisomeric SQMG species or to distinguish the occurrence of a single SQMG with a specific side-chain composition. For this reason, the solid-phase extraction (SPE) protocol described in Materials and Methods was applied and the fraction containing SQMG and SQDG was collected and analyzed by RPLC-ESI-MS [31] (vide infra).

### 2.2. SQDG and SQMG in the Lipid Extract of U. pinnatifida by RPLC-ESI(-)-MS and Tandem MS Analysis

**Figure 3:**
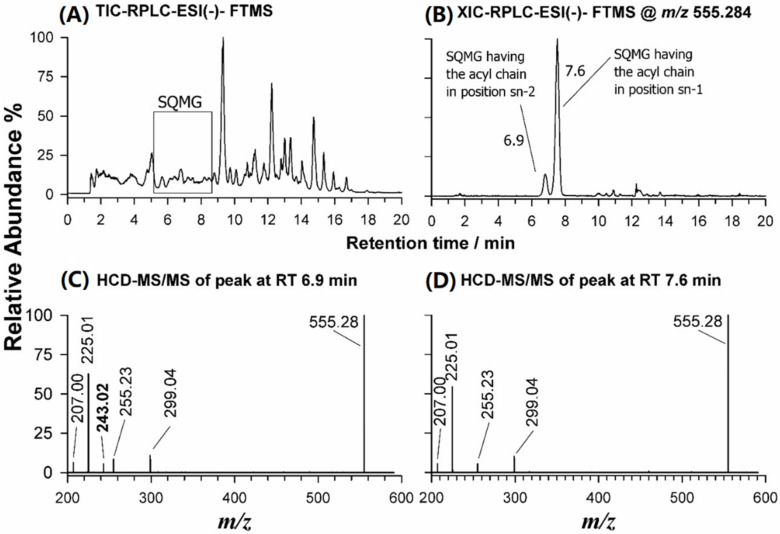
(**A**) Total Ion Current-RPLC-ESI(-)-FTMS chromatogram obtained for the eluate resulting from SPE of the BD extract of *U. pinnatifida*; (**B**) XIC-RPLC-ESI(-)-FTMS chromatogram at *m/z* 555.2828, corresponding to SQMG (16:0); (**C**) ESI(-)-FTMS/MS spectrum obtained for SQMG 0:0/16:0; and (**D**) ESI(-)-FTMS/MS spectrum obtained for SQMG 16:0/0:0.

As shown in the TIC chromatogram reported in Figure 3A, the RPLC-ESI-(-)-FTMS analysis of the eluate obtained from the silica-packed SPE column loaded with the BD extract of *U. pinnatifida* enabled the separation of SQMG on an extended retention time, compared to HILIC performed directly on the lipid extract. Such a purification protocol was found to be useful for harvesting the glycolipids (i.e., SQDG, SQMG, DGDG, and DGMG), whilst most PLs were retained by the SPE silica column.

Due to the occurrence of a sulfonic group in their structure, SQMG like SQDG could be easily detected as deprotonated species ([M-H]^−^) using ESI in negative polarity [27]. As already reported [31,32], it is possible to recognize both SQDG and SQMG species from their tandem mass spectra, thanks to a marker product ion at *m*/*z* 225.01, corresponding to the dehydrated form of the sulfoquinovosyl anionic head with molecular formula [C_6_H_9_O_7_S]^−^. Since each acyl chain generates two product ions, one due to its neutral loss as fatty acid (the neutral loss as ketene is hardly revealed) and the other to the corresponding carboxylate anion, it is possible to infer both the length and unsaturation degree of the fatty acyl chain(s) of SQDG and SQMG [27,31,33].

Furthermore, the tandem MS spectrum of an SQDG precursor ion provides useful information on the *sn*-1/*sn*-2 position of fatty acyl chains, since the loss as fatty acid is favored for the chain located on the *sn*-1 position (see Table 2). As an example, Figure S2A (Supplementary Information) illustrates the MS/MS spectrum of the precursor ion at *m/z* 765.48, recognized as SQDG 30:0, viz. an SQDG having 30 C atoms on both acyl chains, without unsaturations. The lipid class was confirmed by the peak signal at *m/z* 225.01, corresponding to the gas-phase formation of dehydrated sulfoquinovosyl anion, while the R_x_COO^−^ anions were detected at *m/z* 227.20 and 255.23, corresponding, respectively, to myristate, C14:0, and palmitate, C16:0, anions. These fatty acyl chains were confirmed by the two product ions arising from their neutral loss as fatty acids, at *m/z* 537.28 (neutral loss of myristic acid, [M-H-C_13_H_27_COOH]^−^) and 509.24 (neutral loss of palmitic acid, [M-H-C_15_H_31_COOH]^−^). Besides confirming the chain composition, these neutral losses allowed a confident regiochemical assignment, since the product ion at *m/z* 537.28 was more abundant than that at *m/z* 509.24, signifying that the acyl chain 14:0 was placed on the *sn*-1 position of glycerol. The sulfolipid was thus designated as SQDG 14:0/16:0, using the nomenclature described by Liebisch et al. [34] for regioisomeric lipids including a glycerol backbone. The same evaluations were made for other tandem mass spectra of SQDG, all exhibiting two different fatty acyl chains (see Figure S2B–D, Supplementary Information). Looking at the fatty acyl chains, it is evident that the most abundant SQDG displayed the unusual tendency [35,36] of bearing the longest and most unsaturated acyl chain in the *sn*-1 position (see Table 1 and Table S1).

The RPLC separation of the eluate resulting from SPE of the BD extract of *U. pinnatifida* allowed to successfully distinguish both regioisomers of SQMG, when present. As an example, the XIC chromatogram obtained for the *m/z* ratio 555.2828, corresponding to the [M-H]^−^ ion of SQMG (16:0), detected as a single peak after HILIC separation, exhibited two different chromatographic peaks, likely related to the two possible regioisomers (Figure 3B). Their separation was a key step to distinguish the regiochemistry of SQMG species, as emphasized by plots C and D of Figure 3, illustrating the HCD-MS/MS spectra acquired under both RPLC peaks. The main peak signal of both spectra was the typical product ion of SQDG and SQMG, at *m/z* 225.01. Other common product ions were detected at *m/z* 299.04 (neutral loss of the 16:0 acyl chain as fatty acid), at *m/z* 255.23, corresponding to the 16:0 carboxylate, and at *m/z* 207.00, arising from the ion at *m/z* 225.01 upon a further water loss [31]. A key role for the recognition of the two SQMG 16:0 regioisomers was the detection of an *m/z* 243.02 product ion, corresponding to the sulfoquinovosyl anion, [C_6_H_11_O_8_S]^−^, only for the SQMG eluting earlier (6.9 min, see Figure 3C). Indeed, that fragment was previously reported to be generated only for SQMG whose fatty acyl chain is in the *sn*-2 position of glycerol [32] (see Table 2). Consequently, the first eluting regioisomer was assigned as SQMG 0:0/16:0 and the second as SQMG 16:0/0:0. Following the fragmentation rules described so far, 16 SQDG and 6 SQMG were identified in the lipid extracts of *U. pinnatifida*, as summarized in Table S1 of the Supplementary Material; the most abundant SQDG and SQMG species are listed in Table 1.

### 2.3. HILIC-ESI(-)-MS(/MS) and DI-ESI(+)-MS/MS Analysis of DGDG and DGMG in the Lipid Extract of U. pinnatifida

**Figure 4:**
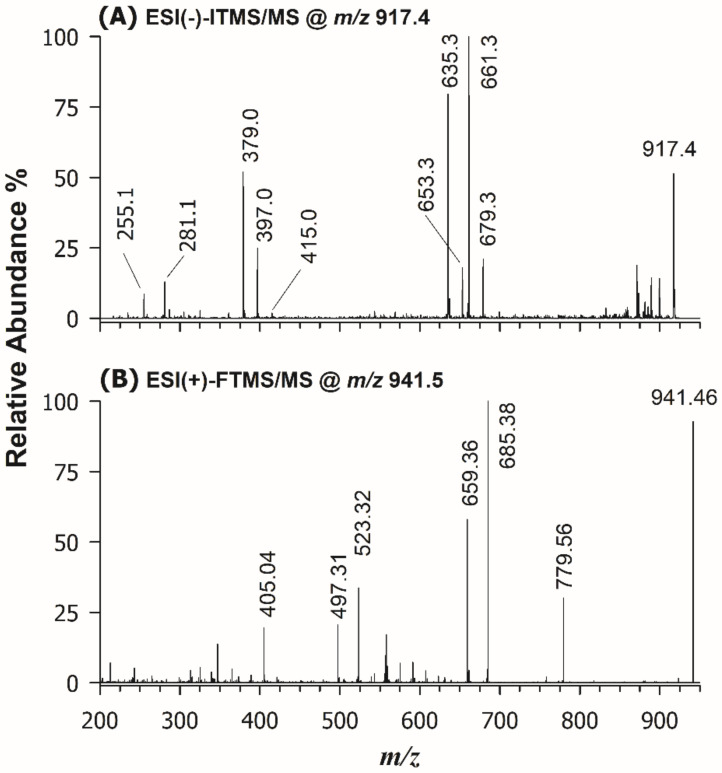
(**A**) ESI(-)-ITMS/MS spectrum, obtained after HILIC separation of the BD extract of *U. pinnatifida*, of the [M-H]^─^ ion detected at *m/z* 917.4; (**B**) ESI(+)-FTMS/MS spectrum, obtained upon direct infusion of the same BD extract after sodium acetate addition, for the [M+Na]^+^ ion detected at *m/z* 941.46. The final assignment inferred from the two spectra was DGDG 16:0/18:1.

The first step of structural characterization of digalactosyl di- and mono-acylglycerols (DGDG and DGMG) was carried out using HILIC retention times and accurate *m/z* ratios for the corresponding ions. The relative content determination was accomplished as mentioned above for SQDG and SQMG. Following the mainstream of this work, tandem MS identification was initially carried out in negative ion mode. As an example, the MS/MS spectrum obtained for the [M-H]^−^ ion detected at *m/z* 917.4 using the linear ion trap is reported in Figure 4A. Typically, when DGDG (or DGMG) are analyzed as deprotonated species, the tandem mass spectrum shows a triad of diagnostic signals arising from the polar head: the product ion at *m/z* 415.0, interpretable as an anion made up of 2 galactose molecules linked to the *sn*-3 position of the glycerol backbone, and two peak signals at *m/z* 397.0 e 379.0, due to the gas-phase sequential losses of one and two water molecules, most likely arising from glycerol and/or galactosyl groups [44]. Notably, chloride ([M+Cl]^−^) and formate ([M+HCOO]^−^) adducts can be formed from DGDG in the ESI source but their tandem mass spectra are as informative as those of [M-H]^─^ ions. Both DGDG and DGMG display three product ions related to each fatty acyl chain, i.e., the carboxylate anion, and ions arising from the FA neutral loss or the ketene neutral loss, which allow identifying the sum composition of both acyl chains. Indeed, the peak signals at *m/z* 255.1 and 281.1, corresponding, respectively, to C16:0 and C18:1 carboxylate anions, and the couples of product ions at *m/z* 661.3/635.3 and 679.3/653.3, corresponding to the neutral losses as FA and ketenes of acyl chains 16:0 and 18:1 can be easily recognized in the MS/MS spectrum reported in Figure 4A. Unfortunately, the neutral losses of FA or ketenes did not allow the empirical regiochemical assignment of DGDG 34:1 because the peak intensities of the corresponding product ions were almost comparable. As demonstrated by Guella et al. [37], the regiochemistry of DGDG and DGMG can be retrieved by considering MS/MS spectra of their [M+Na]^+^ ions (see Table 2). Thus, sodium acetate was added to the BD extract (final concentration 2 mM) and the solution was directly infused (DI) in the Q-Exactive spectrometer, to acquire HCD-FTMS/MS spectra. As an example, the MS/MS spectrum of the sodium adduct for the DGDG 34:1 species, detected at *m/z* 941.46, is illustrated in Figure 4B. In the same spectrum, a diagnostic product ion at *m/z* 405.04, related to the polar head, can be observed [45]; two further diagnostic peak signals, due to the FA neutral losses of the 16:0 and the 18:1 acyl chains, can be seen at *m/z* 685.38 and 659.36. Since the most favored loss as FA is known to occur for the acyl chain located in the *sn*-1 position of glycerol [37], the DGDG could be assigned as 16:0/18:1. Notably, the *m/z* 523.32 and 497.31 product ions detected in the MS/MS spectrum of Figure 4B were related to the neutral loss of a galactosyl unit (162.0 u), respectively, from the *m/z* 685.38 and 659.36 ions. The ion detected at *m/z* 779.56 was also generated from a galactosyl loss, occurring from the precursor ion. By following the fragmentation rules described so far, all main DGDG and DGMG identified in the lipid extract of wakame alga were characterized, and results are listed in Tables S2 and S3 of the Supplementary Information, respectively.

### 2.4. PL and lyso-PL in the Lipid Extract of U. pinnatifida by HILIC-ESI(-)-MS(/MS) Analysis

**Figure 5:**
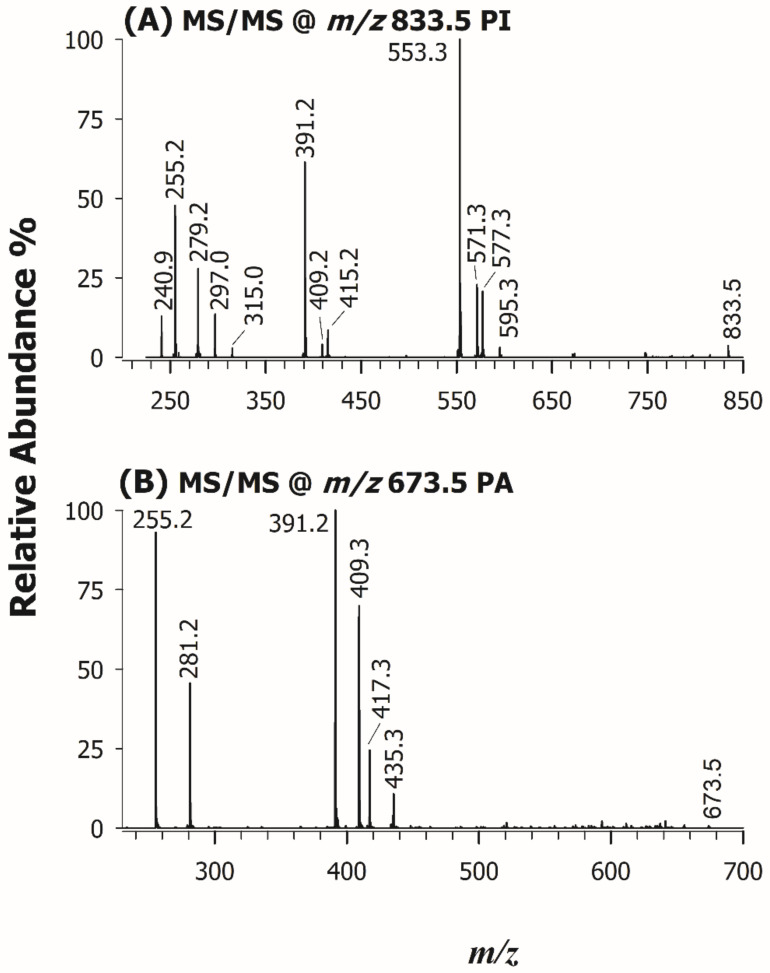
ESI(-)-ITMS/MS spectra of the precursor ions at *m/z* 833.5 (**A**) and 673.5 (**B**), assigned to PI 16:0/18:2 and PA 16:0/18:1, respectively.

Each chromatographic peak/band observed in the HILIC-ESI(-)-FTMS separation was exploited to characterize the most abundant PL species occurring in the lipid extract of *U. pinnatifida*, namely PG, PI, PA, PE, LPE, PC, and LPC (see Figure 1). Accurate *m/z* ratios for all peaks detected in the FTMS spectra averaged under those peaks/bands were employed to infer the sum composition of PL species. As for the structural characterization of PL, despite the high accuracy available for product ions and the absence of a low-mass cut-off, HCD-FTMS/MS spectra were not considered informative from the regiochemical point of view. Indeed, although the generation of carboxylate anions from fatty acyl chains is known to be the main fragmentation route [46], the relative intensity of those ions strongly depends on experimental conditions [47], thus it cannot be exploited for a reliable regiochemical assignment of PL. On the other hand, the acquisition of CID-MS/MS spectra based on the linear ion trap leads to the detection of several intense product ions closely related to acyl chains, namely those resulting from their neutral losses as fatty acids or as ketenes [48]. Taking advantage of the seminal work on PL by Hsu and Turk [43], accomplished using triple quadrupole instruments, it was recognized that the loss as ketene of acyl chains from PC precursor ions (chiefly the [M-CH_3_]^−^ ones, where M is the zwitterionic form of PC) occurs preferentially from the *sn*-2 position of glycerol. The same is true when PA [40], PE [41], PI [39], and PG [38] are subjected to CID fragmentation, with the preferential neutral losses of the acyl chain placed in *sn*-2, as FA or ketene, according to the gas-phase acid/basic character of the precursor ion [49] (see Table 2 and Figure S3). An example of the application of such fragmentation rules to PL extracted from *U. pinnatifida* is reported in Figure 5, showing the ESI(-)-ITMS/MS spectra for ions detected at *m/z* 833.5 Figure 5A and 673.5 Figure 5B, recognized as PI (34:2) and PA (34:1), respectively. The occurrence of two product ions at *m/z* 255.2 and 279.2 in Figure 5A suggested the presence of 16:0 and 18:2 acyl chains. These acyl chains were further confirmed by four peak signals generated as neutral losses as ketenes (at *m/z* 595.3 and 571.3) and as fatty acids (at *m/z* 577.3 and 553.3). The PI class was inferred from the signals at *m/z* 240.9, 297.0, and 315.0, arising from phosphatidylinositol polar head and validated by the three peak signals at *m/z* 391.2, 409.2, and 415.2, resulting from the neutral loss of a dehydrated inositol molecule (C_6_H_10_O_5_, 162.05 Da) from the product ions at *m/z* 553.3, 571.3, and 577.3. The PI regiochemistry was inferred from the relative abundance of peaks referred to FA neutral losses, because in the case of PI this fragmentation is favored from the *sn*-2 position [39]. Since the peak signals at *m/z* 553.3 was much more abundant than that detected at *m/z* 577.3, the phosphatidylinositol species was assigned as PI 16:0/18:2. Similar considerations were applied for the interpretation of the CID-MS/MS spectrum of PA (34:1), reported in Figure 5B; indeed, signals at *m*/*z* 255.2 and 281.2 were assigned as [C_15_H_31_COOH]^−^ and [C_17_H_31_COOH]^−^, respectively, signifying the presence of 16:0 and 18:1 acyl chains. Product ions at *m/z* 391.2 and 417.3, generated from acyl chain losses as fatty acids ([M-H-C_17_H_31_COOH]^−^ and [M-H-C_15_H_31_COOH]^−^, respectively) and those detected at *m/z* 409.3 and 435.3, arising from acyl chain losses as ketenes ([M-H-C_17_H_32_=C=O]^−^ and [M-H- C_15_H_30_=C=O]^−^, respectively) corroborated the presence of the two acyl chains. With regard to regiochemistry, the already known preferential loss of acyl chains as FA from the *sn*-2 position of glycerol in the case of PA [40] was exploited to assign the spectrum to PA 16:0/18:1.

The single acyl chain location on the glycerol backbone of LPC and LPE was recently established employing standard compounds of known regiochemistry [42]. It was shown that the ratio of abundances of product ions detected at *m/z* 196.1 and 214.0 was higher than unity in the case of LPE with the acyl chain located in the *sn*-1 position of glycerol. Likewise, the peak signal detected at nominal *m/z* 224.1 was found to be more abundant than that at *m/z* 242.1 for LPC with the acyl chain located in the *sn*-1 position [42]. It is worth noting that these product ions are related to the polar head and might arise from the neutral loss of the single acyl chain as a ketene (*m/z* 214.0 for LPE and *m/z* 242.1 for LPC) or as fatty acid (*m/z* 196.1 for LPE and *m/z* 224.1 for LPC). Explicative tandem mass spectra of LPC and LPE regioisomers differing only in the acyl chain location are displayed in Figure S4 (Supplementary Information). The complete assignments of PL are reported in the Supplementary Information as Tables S4–S8, PG/LPG, PI, PA, PE/LPE, and PC/LPC, respectively.

### 2.5. Relative Abundance of PL and GL in the Lipid Extract of U. pinnatifida

**Figure 6:**
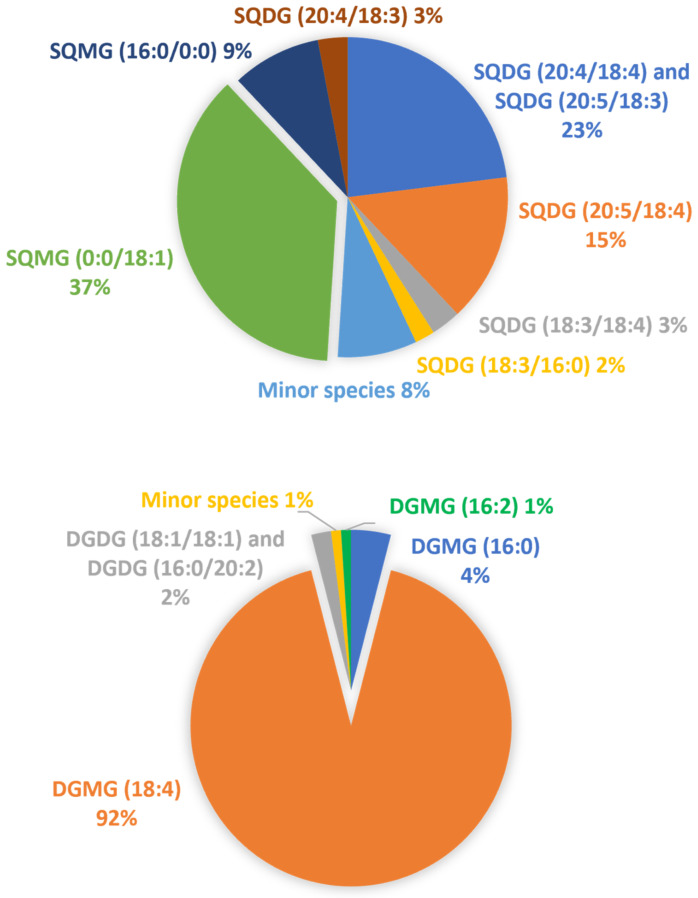
Representation of the relative abundance of the major SQDG (upper panel) and DGDG (lower panel) classes and the relevant lyso-forms (SQMG/DGMG) in the wakame seaweed lipid extracts.

Unlike SQDG, the most abundant PL identified in the lipid extract of *U. pinnatifida* exhibited the longest and most unsaturated acyl chains in the *sn*-2 position. A semi-quantitative comparison of each phospho-, sulfo-, and glycolipid was performed by calculating the relative abundances in the examined extracted sample. A class-related area was computed as the sum of the areas of each detected lipid species from the corresponding extracted ion current (XIC) chromatogram. The relative abundance (r.a.) of each lipid was thus evaluated by its peak area over the area sum of all identified class-related species. Apparently, the most abundant class among them is represented by PI (28%), followed by PG (16%) and corresponding LPG (15%). The fatty acyl chain with 16 carbon atoms (likely corresponding to a palmitoyl chain) was the most abundant saturated one, while among the main monounsaturated and polyunsaturated acyl chains, C18:1, C18:2, and C20:4 were found, in good agreement with previous works on the red algae *Rhodymenia pseudopalmata* [26] and *Gracilaria vermiculophylla* [50]. The occurrence of fatty acyl chains C16:0 and C18:1 in blue-green algae was also demonstrated by Parker et al. [51]. It is worth highlighting that the *U. pinnafitida* lipidome also exhibited the occurrence of odd-numbered acyl chains, namely, C15:0, C17:0, C19:0, and C19:1 (see Tables S1–S8 in Supplementary Information). Although this outcome was in agreement with earlier investigations on marine algae [16,21,22], the existence of microbial contaminations in the analyzed samples cannot be ruled out as an additional source of lipids with odd-numbered side chains. High amounts of myristic and palmitoleic acyl chains were also detected, in agreement with Shanmugam and Palpandi, who investigated the lipid profile of the green alga *Ulva reticulata* [52]. An apparent peculiar result was observed comparing our data with those reported by Takagi et al. [53], who studied the lipidome of plenty of algal specimens, as they reported the presence of minute amounts (r.a. 0.13%) of hexadeca-4Z,7Z,10Z,13Z tetraenoic acid (C16:4 ω3) in the sea mustard. Albeit its occurrence in the wakame algal matrix was also confirmed by Ishihara et al. [54] and Schlotterbeck et al. [22], no presence of the 16:4 chain was found in our samples. This relatively short and highly unsaturated acyl chain might occur in neutral lipids (e.g., glycerolipids), or can exist as one of the free fatty acids, both not investigated in this work. For glycolipids, semi-quantitative data can be obtained since the ratio is calculated within the same lipid class being the ionization yield less affected by their acyl chain properties. As already mentioned, the r.a. of each lipid species within a specific class, was estimated from the corresponding XIC chromatogram, then the sum of all peak areas for compounds detected in the class was calculated. In Figure 6 are depicted the r.a. of SQDG (upper panel) and DGDG (lower panel) classes and the relevant *lyso*-forms (SQMG/DGMG) in the wakame seaweed lipid extracts. Concerning the fatty acyl chains of SQDG in brown algae, our findings are in good agreement with those of Khotimchenko [55] because FA 16:0, 18:1, 18:2, and 18:3 were found to be predominant within this sulfolipid class. The abundance of fatty acyl chains C16:0 and C18:1 was already demonstrated in the DGDG profile of brown algae [56,57]. It is worth mentioning the prevailing detection of DGMG 18:4 that agreed with previous findings [22] and provided compelling evidence of this fatty acyl chain in the wakame seaweed.

## 3. Materials and Methods

### 3.1. Chemicals and Samples

LC-MS grade water, methanol, propan-2-ol and acetonitrile (ACN), HPLC grade chloroform, reagent grade formic acid, ammonium formate, and sodium acetate were purchased from Sigma Aldrich (Milan, Italy). Various wakame seaweed samples were bought, as dried chunks, from local grocery stores.

### 3.2. LC-MS Instrumentation and Operating Conditions

Two Ultimate 3000 UHPLC chromatographic systems, coupled by a heated electrospray source ionization (HESI) either to a Velos Pro linear ion trap (LIT) mass spectrometer (Thermo Scientific, Waltham, MA, USA) or to a Q-Exactive quadrupole-Orbitrap™ mass spectrometer (Thermo Scientific, Waltham, MA, USA) were employed. The analyses performed using the Velos Pro were labeled by the tag “IT” (ion trap), while analyses made via Q-Exactive were branded as “FT” (Fourier Transform). The use of both mass spectrometers aimed at exploiting the high mass accuracy and resolution proved by the Orbitrap analyzer and, in the case of MS/MS acquisitions, to have a more informative fragmentation pattern using both the higher-energy collisional dissociation regime available in the HCD cell of the Q-Exactive spectrometer and the low energy collisional induced/activated dissociation (CID/CAD) occurring in the Velos Pro one.

HILIC separation was performed on a Ascentis Express HILIC column packed with silica core-shell particles (150 × 2.1 mm ID, 2.7 µm particle size, 1.7 µm core size) and preceded by an Ascentis Express HILIC (5 × 2.1 mm ID) security guard cartridge (Supelco, Bellefonte, PA, USA) at 25 °C and using a flow rate of 300 μL/min; a binary gradient was used, composed of water (eluent A) and acetonitrile (eluent B), both containing 0.1% (*v*/*v*) formic acid and 2.5 mM ammonium formate [58]. The gradient elution program was the following [55]: 0–5 min linear from 3% to 12% (*v*/*v*) solvent B; 5–10 min isocratic at 12% (*v*/*v*) solvent B; 10–11 min linear from 12% to 19% (*v*/*v*) solvent B; 11–20 min linear from 19% to 30% (*v*/*v*) solvent B; 20–22 min linear from 30% to 50% (*v*/*v*) solvent B; 22–28 min isocratic at 50% (*v*/*v*) solvent B; 28–30 min linear gradient from 50% to 3% (*v*/*v*) solvent B, followed by 5 min equilibration time [56].

RPLC separations were based on a Ascentis Express C18 column also packed with core-shell particles (150 × 2.1 mm ID, 2.7 µm particle size, 1.7 µm core size) and equipped with an Ascentis Express C18 (5 × 2.1 mm ID) security guard cartridge (Supelco, Bellefonte, PA, USA) at 40 °C, using a flow rate of 200 μL/min. RPLC separations were performed using a binary gradient [57] based on water/propan-2-ol (60/40, *v*/*v*) (eluent A) and methanol/propan-2-ol (60/40, *v*/*v*) (eluent B), both containing 0.2% (*v*/*v*) formic acid and 10 mM ammonium formate. The gradient elution program was the following: 0–7 min linear from 40% to 80% (*v*/*v*) solvent B; 7–13 min isocratic at 80% (*v*/*v*) solvent B; 13–18 min linear from 80% to 90% (*v*/*v*) solvent B; 18–21 min isocratic at 90% (*v*/*v*) solvent B; 21–26 min linear from 90% to 100% (*v*/*v*) solvent B; 26–29 min isocratic at 100% (*v*/*v*) solvent B; 29–31 min linear from 100% to 40% (*v/v*) solvent B, followed by 5 min equilibration time.

The following values were adopted for the Q-Exactive heated ESI source and ion optics parameters: sheath gas flow rate, 35 arbitrary units (a.u.); auxiliary gas flow rate, 15 a.u.; spray voltage, −2.5 kV (negative polarity), or +3.5 kV (positive polarity); capillary temperature, 320 °C; S-lens radio frequency (R.F.) level, 100 a.u. in negative polarity and 60 a.u. in positive polarity. MS full scan acquisitions were performed in negative ion mode, in the *m/z* range 150–2000, after setting a mass resolving power of 140,000 (at *m/z* 200). During MS measurements, the Orbitrap fill time was set to 200 ms and the automatic gain control (AGC) level to 3 × 10^6^. The Q-Exactive spectrometer was calibrated once in two days and mass accuracy ranged between 0.11–0.16 ppm. in positive polarity and between 0.41–0.44 ppm. in negative polarity. FTMS/MS acquisitions were performed both in positive (only by direct infusion, DI, mode) and in negative ion mode on targeted precursor ions using an isolation window of 1.0 *m/z* centered on the corresponding exact *m/z* ratios, resolving power of 35,000 (at *m/z* 200), a fill time of 100 ms and an AGC level of 2 × 10^5^; normalized collision energy (NCE) was 35%.

The following values were adopted for the Velos Pro heated ESI source and ion optics parameters: sheath gas flow rate, 35 a.u.; auxiliary gas flow rate, 5 a.u.; spray voltage, −2.5 kV (negative polarity); capillary temperature, 350 °C; S-lens RF level, 64.20 a.u. MS *full scan* acquisitions were performed in negative ion mode, in the *m/z* range 110–2000. During MS measurements, the Velos Pro fill time was set to 10 ms and the AGC level to 3 × 10^4^. MS/MS acquisitions were performed in negative ion mode on targeted precursor ions using an isolation window of 1 *m/z* centered on the corresponding exact *m/z* ratios, an ion trap fill time of 50 ms, and an AGC level of 1 × 10^4^; NCE was set as 35% for MS/MS.

For both instruments a source-induced fragmentation (sid) at 40 eV was exploited to enhance the formation of [M-CH_3_]^−^ ions from adducts with formate characteristic of phospholipids (PL) having a choline moiety in their polar head, for instance, phosphocholines (PC) and lyso-PC (LPC) [59]. The control of LC-MS instrumentation and the first data processing was performed by the Xcalibur software 2.2 SP1.48 (Thermo Scientific, Waltham, MA, USA). MS raw data were imported, further elaborated, and finally turned into figures by the SigmaPlot 14.5 software (Systat Software, Inc., London, UK). The ChemDraw Pro 8.0.3 software (CambridgeSoft Corporation, Cambridge, MA, USA) was employed to draw chemical structures.

### 3.3. Extraction of Lipids

Lipid extraction was performed by following the Bligh & Dyer (BD) protocol [60]. Briefly, a sample of dried seaweed (about 0.2 g) was placed in a test tube, soaked in ca. 10 mL of LC-MS grade H_2_O and left for 20 min to rehydrate it and to remove the salt excess. Then, water was thrown away and the sample was placed in a clean test tube along with 3 mL of a CH_3_OH/CHCl_3_ 2:1 (*v*/*v*) mixture and 800 µL of water. The whole system was vortexed and stored at room temperature for one hour. Afterward, 1 mL of CHCl_3_ and 1 mL of H_2_O were added to the sample; the mixture was vortexed once again and centrifuged for 10 min at 3000× *g*. The chloroform phase was recovered and dried under N_2_ flow, then the resulting pellet was dissolved in 100 μL of CH_3_OH/CHCl_3_ (1:1, *v*/*v*), ready to be injected in LC-MS systems.

### 3.4. Solid-Phase Extraction of Lipid Extracts

For the detailed analysis of SQDG and their lyso-forms, the BD extract was subjected to solid-phase extraction (SPE) on a column consisting of a glass Pasteur pipette packed with silica particles. After testing various conditions for SPE column conditioning/washing and elution (type and volume ratio of solvents), the following protocol was set up. The silica packing (200 mg/column) was conditioned with 1 mL of ACN (2×); dried BD extract was then dissolved in 1 mL of ACN and loaded. The column was washed again with 1 mL of acetonitrile and the elution was carried out with 1 mL of ACN/H_2_O 80:20 (*v*/*v*) containing 0.1% formic acid. The purified product was dried under N_2_ and dissolved in 100 μL of CH_3_OH for successive RPLC-ESI(-)-FTMS(/MS) analysis.

## 4. Conclusions

The careful combination of HILIC or RPLC with low- or high-resolution single/tandem mass spectrometry enabled an unprecedented characterization of the lipidome of the edible seaweed *U. pinnatifida*. More than 200 polar lipids, including 16 SQDG, 6 SQMG, 12 DGDG, 5 DGMG, among glycolipids (GL), and 29 PG, 8 LPG, 19 PI, 14 PA, 19 PE, 8 PE, 38 PC, and 27 LPC, as phospholipids (PL), were identified. The length and the amount of unsaturation of fatty acyl chains, alongside their regiochemistry (*sn*-1/*sn*-2 assignment), were established, with just a few exceptions related to low abundance species. While the C16:0 was the most abundant saturated acyl chain, the monounsaturated C18:1 and the polyunsaturated C18:2 and C20:4 were the prevailing ones. Unlike the investigated GL, bearing the longest and most unsaturated acyl chains in the *sn*-1 position of glycerol, this type of fatty acyl chain was mostly observed in the *sn*-2 position in the case of PL. This outcome may be especially useful to appropriately implement lipase-catalyzed bioconversion of PL and GL extracted from wakame.

## Figures and Tables

**Table 1:** List of the most abundant PL and GL species identified in the edible alga wakame ^a^.

#	Accurate *m/z*	Mass Error (ppm.)	SQDG (sn-1/sn-2)	Adduct	Molecular Formula	Relative Abundance (%)
**1**	815.4978	−0.9	18:3/16:0	[M-H]^−^	[C_43_H_75_O_12_S]^−^	22.4
**2**	817.5143	0.2	18:2/16:0		[C_43_H_77_O_12_S]^−^	16.2
**3**	819.5322	2.9	18:1/16:0		[C_43_H_79_O_12_S]^−^	48.2
			**SQMG (*sn*-1/*sn*-2)**			
**4**	555.2820	−4.5	16:0/0:0	[M-H]^−^	[C_25_H_47_O_11_S]^−^	65.0
**5**			0:0/16:0			12.0
			**DGDG (*sn*-1/*sn*-2)**			
**6**	967.6330	0.2	18:1/18:1; 16:0/20:2	[M+Na]^+^	[C_51_H_92_O_15_Na]^+^	70
**7**	969.6483	−0.2	18:0/18:1		[C_51_H_94_O_15_Na]^+^	15
			**DGMG** ^b^			
**8**	673.3455	2.1	18:4	[M-H]^−^	[C_33_H_53_O_14_]^−^	93
	709.3223	2.1		[M+^35^Cl]^−^	[C_33_H_54_O_14_Cl]^−^	
	711.3170	−1.1		[M+^37^Cl]^−^	[C_33_H_54_O_14_Cl]^−^	
**9**	685.3219	1.6	16:2	[M+^35^Cl]^−^	[C_31_H_54_O_14_Cl]^−^	1.2
			**PG (*sn*-1/*sn*-2)**			
**10**	719.4895	3.6	16:0/16:1; 18:1_14:0	[M-H]^−^	[C_38_H_72_O_10_P]^−^	13.67
**11**	745.5030	0.7	16:0/18:2; 18:1/16:1		[C_40_H_74_O_10_P]^−^	20.47
**12**	747.5177	−0.7	16:0/18:1		[C_40_H_76_O_10_P]^−^	38.09
			**LPG** ^b^			
**13**	481.2571	−0.2	16:1	[M-H]^−^	[C_22_H_42_O_9_P]^−^	28.89
**14**	483.2730	0.4	16:0		[C_22_H_44_O_9_P]^−^	20.80
**15**	507.2729	0.2	18:2		[C_24_H_44_O_9_P]^−^	16.87
**16**	509.2883	−0.4	18:1		[C_24_H_46_O_9_P]^−^	24.34
			**PI (*sn*-1/*sn*-2)**			
**17**	833.5197	1.3	16:0/18:2	[M-H]^−^	[C_43_H_78_O_13_P]^−^	18.78
**18**	835.5349	0.8	16:0/18:1		[C_43_H_80_O_13_P]^−^	69.69
			**PA (*sn*-1/*sn*-2)**			
**19**	645.4516	2.3	16:0/16:1; 14:0/18:1	[M-H]^−^	[C_35_H_66_O_8_P]^−^	12.80
**20**	695.4668	1.6	16:0/20:4; 18:2/18:2		[C_39_H_68_O_8_P]^−^	12.88
**21**	743.4671	1.9	20:4/20:4		[C_43_H_68_O_8_P]^−^	47.85
			**PE (*sn*-1/*sn*-2)**			
**22**	686.4792	3.8	16:1/16:1	[M-H]^−^	[C_37_H_69_NO_8_P]^−^	7.11
**23**	688.4929	0.9	16:0/16:1; 18:1/14:0		[C_37_H_71_NO_8_P]^−^	63.02
**24**	714.5069	−1.4	18:1/16:1		[C_39_H_73_NO_8_P]^−^	9.68
**25**	738.5092	1.8	16:0/20:4		[C_41_H_73_NO_8_P]^−^	6.06
**26**	786.5100	2.7	20:4/20:4		[C_45_H_73_NO_8_P]^−^	5.52
			**LPE (*sn*-1/*sn*-2)**			
**27**	500.2797	2.8	20:4/0:0	[M-H]^−^	[C_25_H_43_NO_7_P]^−^	65.07
			**PC (*sn*-1/*sn*-2)**			
**28**	774.5302	1.4	14:0/18:2; 16:0/16:2;	[M+HCOO]^−^	[C_41_H_77_NO_10_P]^−^	9.45
			16:1/16:1; 19:2_13:0			
**29**	802.5614	1.2	16:0/18:2		[C_43_H_81_NO_10_P]^−^	34.05
**30**	826.5598	−0.7	16:0/20:4; 18:2/18:2		[C_45_H_81_NO_10_P]^−^	17.73
**31**	850.5580	−2.8	20:4/18:2		[C_47_H_81_NO_10_P]^−^	12.05
			**LPC (*sn*-1/*sn*-2)**			
**32**	504.3113	3.4	18:2/0:0	[M-CH_3_]^−^	[C_25_H_47_NO_7_P]^−^	11.17
**33**	564.3323	2.8	18:2/0:0	[M+HCOO]^−^	[C_27_H_51_NO_9_P]^−^	23.71
**34**	588.3323	2.7	20:4/0:0		[C_29_H_51_NO_9_P]^−^	22.32

^a^ A more extended list of PL and GL species is provided in the Supplementary Information. ^b^ No regiochemical assignment is reported.

**Table 2:** Summary of class-related regiochemical assignment by ESI-MS/MS of phospholipids, sulfolipids, and glycolipids along with their lyso forms identified in the lipid extract of wakame brown alga (*U. pinnatifida*).

Lipid Class	Precursor Ion	Diagnostic Product Ion for Regiochemical Assignment: *sn*-1/*sn*-2	Comparison between Peak Signal Intensity	Ref.
SQDG	[M-H]^−^	[M-H-R_1_COOH]^−^; [M-H-R_2_COOH]^−^	[M-H-R_1_COOH]^−^ > [M-H-R_2_COOH]^−^	[31]
SQMG	[M-H]^−^	*m/z* 243.0, [C_6_H_11_O_8_S]^−^	Product ion is generated if the FA is at *sn*-2	[32]
DGDG	[M+Na]^+^	[M+Na-R_1_COOH]^+^; [M+Na-R_2_COOH]^+^	[M+Na-R_1_COOH]^+^ > [M+Na-R_2_COOH]^+^	[37]
PG	[M-H]^−^	[M-H-R_1_COOH]^−^; [M-H-R_2_COOH]^−^	[M-H-R_2_COOH]^−^ > [M-H-R_1_COOH]^−^	[38]
PI	[M-H]^−^	[M-H-R_1_COOH]^−^; [M-H-R2COOH]^−^	[M-H-R_2_COOH]^−^ > [M-H-R_1_COOH]^−^	[39]
PA	[M-H]^−^	[M-H-R_1_COOH]^−^; [M-H-R_2_COOH]^−^	[M-H-R_2_COOH]^−^ > [M-H-R_1_COOH]^−^	[40]
PE	[M-H]^−^	[M-H-R’_1_=C=O]^−^; [M-H-R’_2_=C=O]^−^	[M-H-R’_2_=C=O]^−^ > [M-H-R’_1_=C=O]^−^	[41]
LPE	[M-H]^−^	*m/z* 196.1, [M-H-RCOOH]^−^;	If RCOOH is in *sn*-1: [M-H-RCOOH]^−^ > [M-H-R’=C=O]^−^ else viceversa	[42]
		*m/z* 214.1, [M-H-R’=C=O]^−^		
PC	[M-CH_3_]^−^,	[M-CH_3_-R’_1_=C=O]^−^; [M-CH_3_-R’_2_=C=O]^−^	[M-CH_3_-R’_2_=C=O]^−^ > [M-CH_3_-R’_1_=C=O]^−^	[43]
	[M+HCOO]^−^			
LPC	[M-CH_3_]^−^,	*m/z* 224.1, [M-CH_3_-RCOOH]^−^	If RCOOH is in *sn*-1: [M-CH_3_-RCOOH]^−^ > [M-CH_3_-R’=C=O]^−^ else viceversa	[42]
	[M+HCOO]^−^	*m/z* 242.1, [M-CH_3_-R’=C=O]^−^		

## Data Availability

The authors confirm that most of the data supporting the findings of this study are available within the article and its Supplementary Materials. Raw data are available from the corresponding authors (C.D.C., T.R.I.C.) on request.

## Supplementary Materials

The following are available online, Table S1: List of SQDG and SQMG identified as deprotonated molecules, [M-H]^−^, in the edible brown alga wakame by RPLC-ESI(-)-HCDMS/MS. The most abundant species are emphasized by bold fonts. Table S2: List of DGDG identified as sodiated adducts, [M+Na]^+^, in the edible brown alga wakame by DI-ESI(+)-HCDMS/MS. The most abundant species are reported in bold. Table S3: List of DGMG identified in negative ion mode in the edible brown alga wakame by HILIC-ESI(-)-HCDMS/MS. The most abundant species are in bold. Table S4: List of PG and lyso-forms identified as deprotonated molecules, [M-H]^−^, in the edible brown alga wakame by HILIC-ESI(-)CIDMS/MS. The most abundant species are in bold. Table S5: List of PI identified as deprotonated molecules, [M-H]^−^, in the edible brown alga wakame by HILIC-ESI(-)-CID/MS/MS. The most abundant species are in bold. Table S6: List of PA identified as deprotonated molecules, [M-H]^−^, in the edible brown alga wakame by HILIC-ESI(-)-CID/MS/MS. The most abundant species are in bold. Table S7: List of PE and LPE identified as deprotonated molecules, [M-H]^−^, in the edible brown alga wakame by HILIC-ESI(-)-CID/MS/MS. The most abundant species are in bold. Table S8: List of PC and LPC identified in negative ion mode in the edible brown alga wakame by HILIC-ESI(-)-CID/MS/MS. The most abundant species are in bold. Figure S1: Molecular structures of the investigated lipid classes. Sulfoquinovosyl diacylglycerols (SQDG), digalactosyldiacylglycerols (DGDG), phosphatidylglycerols (PG), phosphatidylinositols (PI), phosphatidic acids (PA), phosphatidylethanolamines (PE), and phosphatidylcholines (PC). RPLC-ESI(-)-FTMS/MS spectra of the ions at (**A**) *m/z* 765.48, SQDG 14:0/16:0, (**B**) *m/z* 819.53, SQDG 18:01/16:0, (**C**) *m/z* 831.53, SQDG 19:2/16:0, and (**D**) *m/z* 859.47, SQDG 20:5/18:4. Figure S3: HILIC-ESI(-)-FTMS/MS spectra of the ions at (**A**) *m/z* 747.5, PG 16:0/18:1, (**B**) *m/z* 509.3, LPG 18:1, (**C**) *m/z* 742.5, PC 16:0/18:2, and (**D**) *m/z* 738.5, PE 18:0/20:4. Figure S4: HILIC-ESI(-)-ITMS/MS spectra averaged under the four chromatographic peaks related to the ion at *m/z* 478.3, identified as (**A**) LPE 18:1/0:0, (**B**) LPE 0:0/18:1, (**C**) LPC 16:1/0:0, and (**D**) LPC 0:0/16:1.

## Sample Availability

Samples of the compound are not available from the authors.
