# Supplementary material for: Lipidomics of the Edible Brown Alga Wakame (Undaria pinnatifida) by Liquid Chromatography Coupled to Electrospray Ionization and Tandem Mass Spectrometry
Source: Molecules. 2021 Jul 24;26(15):4480. doi: 10.3390/molecules26154480 (PMC8348742; doi:10.3390/molecules26154480)
Supplement: Supplementary file 1 [file molecules-26-04480-s001.zip › molecules-1289213-supplementary.pdf]

Supplementary Material

Lipidomics of the Edible Brown Alga Wakame (*Undaria pinnatifida*) by Liquid Chromatography Coupled to Electrospray Ionization and Tandem Mass Spectrometry

Davide Coniglio [1], Mariachiara Bianco [1], Giovanni Ventura [1], Cosima D. Calvano [2,3,*], Ilario Losito [1,2] and Tommaso R. I. Cataldi [1,2,*]

[1] Dipartimento di Chimica, Università degli Studi di Bari Aldo Moro, via Orabona 4, 70126 Bari, Italy; davide.coniglio@uniba.it (D.C.); mariachiara.bianco@uniba.it (M.B.); giovanni.ventura@uniba.it (G.V.); ilario.losito@uniba.it (I.L.)

[2] Centro Interdipartimentale SMART, Università degli Studi di Bari Aldo Moro, via Orabona 4, 70126 Bari, Italy

[3] Dipartimento di Farmacia-Scienze del Farmaco, Università degli Studi di Bari Aldo Moro, via Orabona 4, 70126 Bari, Italy

* Correspondence: cosimadamiana.calvano@uniba.it (C.D.C.); tommaso.cataldi@uniba.it (T.R.I.C.)

Table S1. List of SQDG and SQMG identified as deprotonated molecules, [M-H]⁻, in the edible brown alga wakame by RPLC-ESI(-)-HCDMS/MS. The most abundant species are emphasized by bold fonts.

#	Accurate *m/z*	Mass Error (ppm)	SQDG (*sn*-1/ *sn*-2)	Molecular Formula [M-H]⁻	Relative Abundance (%)
1	765.4840	1.6	14:0/16:0	$[C_{39}H_{73}O_{12}S]^-$	2.9
2	789.4843	1.9	14:0/18:2	$[C_{41}H_{73}O_{12}S]^-$	2.5
3	791.5001	2.0	16:1 /16:0	$[C_{41}H_{75}O_{12}S]^-$	2.8
**4**	**815.4978**	**−0.9**	**18:3/16:0**	$[C_{43}H_{75}O_{12}S]^-$	**22.4**
**5**	**817.5143**	**0.2**	**18:2/16:0**	$[C_{43}H_{77}O_{12}S]^-$	**16.2**
**6**	**819.5322**	**2.9**	**18:1/16:0**	$[C_{43}H_{79}O_{12}S]^-$	**48.2**
7	835.4692	2.4	18:3/18:4	$[C_{45}H_{71}O_{12}S]^-$	0.7
8	837.4830	0.2	18:3/18:3; 18:2/18:4	$[C_{45}H_{73}O_{12}S]^-$	0.9
9	859.4689	2.0	20:5/18:4	$[C_{47}H_{71}O_{12}S]^-$	1.2
10	861.4837	1.0	20:4/18:4; 20:5/18:3	$[C_{47}H_{73}O_{12}S]^-$	2.0
11	863.4991	0.7	20:4/18:3	$[C_{47}H_{75}O_{12}S]^-$	0.08
			**SQMG (*sn*-1/ *sn*-2)**		
**1**	**555.2820**	**−4.5**	**16:0/0:0**	$[C_{25}H_{47}O_{11}S]^-$	**65.0**
**2**			**0:0/16:0**		**12.0**
3	577.2684	−0.7	18:3/0:0	$[C_{27}H_{49}O_{11}S]^-$	5.6
4			0:0/18:3		1.2
5	581.3000	−0.2	18:1/0:0	$[C_{27}H_{49}O_{11}S]^-$	8.0
6			0:0/18:1		8.2

Table S2. List of DGDG identified as sodiated adducts, $[M+Na]^+$, in the edible brown alga wakame by DI-ESI(+)-HCDMS/MS. The most abundant species are reported in bold.

#	Accurate *m/z*	Mass Error (ppm)	DGDG (*sn*-1/*sn*-2)	Molecular Formula $[M+Na]^+$	Relative Abundance (%)
1	911.5702	0.0	16:1/16:1; 16:0/16:2	$[C_{47}H_{86}O_{15}Na]^+$	0.32
2	913.5860	0.1	16:0/16:1	$[C_{47}H_{86}O_{15}Na]^+$	0.73
3	935.5702	0.0	16:0/18:4	$[C_{49}H_{84}O_{15}Na]^+$	0.01
4	937.5860	0.1	16:0/18:3	$[C_{49}H_{86}O_{15}Na]^+$	0.35
5	939.6016	0.1	16:0/18:2	$[C_{49}H_{88}O_{15}Na]^+$	3.10
6	941.6171	−0.1	16:0/18:1	$[C_{49}H_{90}O_{15}Na]^+$	0.77
7	959.5702	0.0	18:3/18:3	$[C_{51}H_{84}O_{15}Na]^+$	8.70
8	965.6172	0.0	18:2/18:1	$[C_{51}H_{90}O_{15}Na]^+$	0.05
**9**	**967.6330**	**0.2**	**18:1/18:1; 16:0/20:2**	$[C_{51}H_{92}O_{15}Na]^+$	**70.00**
**10**	**969.6483**	**−0.2**	**18:0/18:1**	$[C_{51}H_{94}O_{15}Na]^+$	**15.00**

Table S3. List of DGMG identified in negative ion mode in the edible brown alga wakame by HILIC-ESI(-)-HCDMS/MS. The most abundant species are in bold.

#	Accurate *m/z*	Mass Error (ppm)	DGMG[b]	Adduct	Molecular Formula of the Adduct	Relative Abundance (%)
1	653.3761	1.1	16:0	$[M-H]^-$	$[C_{31}H_{57}O_{14}]^-$	4.20
	689.3527	0.9		$[M+^{35}Cl]^-$	$[C_{31}H_{58}O_{14}Cl]^-$	
	**673.3455**	**2.1**		**$[M-H]^-$**	**$[C_{33}H_{53}O_{14}]^-$**	
**2**	**709.3223**	**2.1**	**18:4**	**$[M+^{35}Cl]^-$**	**$[C_{33}H_{54}O_{14}Cl]^-$**	**93.00**
	**711.3170**	**−1.1**		**$[M+^{37}Cl]^-$**	**$[C_{33}H_{54}O_{14}Cl]^-$**	
**3**	**685.3219**	**1.6**	**16:2**	**$[M+^{35}Cl]^-$**	**$[C_{31}H_{54}O_{14}Cl]^-$**	**1.20**
4	733.3663	1.5	19:4	$[M+HCOO]^-$	$[C_{35}H_{57}O_{16}]^-$	0.22
5	749.3513	−1.1	21:5	$[M+^{35}Cl]^-$	$[C_{36}H_{58}O_{14}Cl]^-$	0.13

[b] No regiochemical assignment was performed.

Table S4. List of PG and lyso-forms identified as deprotonated molecules, $[M-H]^-$, in the edible brown alga wakame by HILIC-ESI(-)CIDMS/MS. The most abundant species are in bold.

#	Accurate *m/z*	Mass Error (ppm)	PG (*sn*-1/*sn*-2)	Molecular Formula $[M-H]^-$	Relative Abundance (%)
1	717.4734	3.1	16:1/16:1; 14:0/18:2	$[C_{38}H_{70}O_{10}P]^-$	1.31
**2**	**719.4895**	**3.6**	**16:0/16:1; 18:1_14:0**	$[C_{38}H_{72}O_{10}P]^-$	**13.67**
3	731.4872	0.4	15:0/18:2; 15:1_18:1; 17:1/16:1; 17:2/16:0	$[C_{39}H_{72}O_{10}P]^-$	0.10
4	733.5031	0.8	16:0/17:1; 15:0/18:1; 19:1/14:0	$[C_{39}H_{74}O_{10}P]^-$	0.41
5	743.4889	2.7	18:3/16:0; 18:2/16:1	$[C_{40}H_{72}O_{10}P]^-$	7.82
**6**	**745.5030**	**0.7**	**16:0/18:2; 18:1/16:1**	$[C_{40}H_{74}O_{10}P]^-$	**20.47**
**7**	**747.5177**	**−0.7**	**16:0/18:1**	$[C_{40}H_{76}O_{10}P]^-$	**38.09**
8	765.4691	−2.7	18:3/18:3; 20:5/16:1	$[C_{42}H_{70}O_{10}P]^-$	3.34
9	767.4859	−1.3	18:3/18:2; 20:4/16:1; 20:5_16:0	$[C_{42}H_{72}O_{10}P]^-$	3.79
10	769.5017	−1.0	18:2/18:2; 16:0_20:4	$[C_{42}H_{74}O_{10}P]^-$	2.87
11	773.5321	−2.2	18:1/18:1; 18:2/18:0	$[C_{42}H_{78}O_{10}P]^-$	3.95
12	775.5505	1.3	18:0/18:1	$[C_{42}H_{80}O_{10}P]^-$	3.46
13	793.5007	−2.3	20:4/18:2; 20:5/18:1	$[C_{44}H_{74}O_{10}P]^-$	0.40
14	795.5204	2.8	20:4/18:1	$[C_{44}H_{76}O_{10}P]^-$	0.32
			**LPG**[a]		
**1**	**481.2571**	**−0.2**	**16:1**	$[C_{22}H_{42}O_9P]^-$	**28.89**
**2**	**483.2730**	**0.4**	**16:0**	$[C_{22}H_{44}O_9P]^-$	**20.80**
3	505.2579	1.4	18:3	$[C_{24}H_{42}O_9P]^-$	6.66
**4**	**507.2729**	**0.2**	**18:2**	$[C_{24}H_{44}O_9P]^-$	**16.87**
**5**	**509.2883**	**−0.4**	**18:1**	$[C_{24}H_{46}O_9P]^-$	**24.34**
6	511.3050	1.8	18:0	$[C_{24}H_{48}O_9P]^-$	0.83
7	531.2738	1.9	20:4	$[C_{26}H_{44}O_9P]^-$	1.26
8	533.2896	2.1	20:3	$[C_{26}H_{46}O_9P]^-$	0.36

[a] No regiochemical assignment was performed.

Table S5. List of PI identified as deprotonated molecules, $[M-H]^-$, in the edible brown alga wakame by HILIC-ESI(-)-CID/MS/MS. The most abundant species are in bold.

#	Accurate *m*/*z*	Mass Error (ppm)	PI (*sn*-1/*sn*-2)	Molecular Formula [M-H]⁻	Relative Abundance (%)
1	805.4890	2.1	14:0/18:2; 16:2_16:0; 16:1/16:1	$[C_{41}H_{74}O_{13}P]^-$	0.67
2	807.5033	0.5	16:0/16:1; 14:0/18:1	$[C_{41}H_{76}O_{13}P]^-$	3.07
3	821.5213	3.3	15:0/18:1	$[C_{42}H_{78}O_{13}P]^-$	0.70
4	831.5040	1.3	16:0/18:3	$[C_{43}H_{76}O_{13}P]^-$	2.88
**5**	**833.5197**	**1.3**	**16:0/18:2**	$[C_{43}H_{78}O_{13}P]^-$	**18.78**
**6**	**835.5349**	**0.8**	**16:0/18:1**	$[C_{43}H_{80}O_{13}P]^-$	**69.69**
7	851.5621	−4.0	16:0/19:0	$[C_{44}H_{84}O_{13}P]^-$	0.11
8	857.5162	−2.8	18:2/18:2; 16:0/20:4; 18:3_18:1	$[C_{45}H_{78}O_{13}P]^-$	2.07
9	859.5332	−1.2	18:2/18:1; 16:0/20:3	$[C_{45}H_{80}O_{13}P]^-$	0.61
10	861.5489	−1.2	18:0/18:2; 18:1/18:1; 16:0/20:2	$[C_{45}H_{82}O_{13}P]^-$	0.62
11	863.5654	−0.1	18:0/18:1	$[C_{45}H_{84}O_{13}P]^-$	0.81

Table S6. List of PA identified as deprotonated molecules, $[M-H]^-$, in the edible brown alga wakame by HILIC-ESI(-)-CID/MS/MS. The most abundant species are in bold.

#	Accurate *m/z*	Mass Error (ppm)	PA (*sn*-1/*sn*-2)	Molecular Formula $[M-H]^-$	Relative Abundance (%)
1	643.4358	2.2	16:1/16:1; 14:0/18:2	$[C_{35}H_{64}O_8P]^-$	2.61
**2**	**645.4516**	**2.3**	**16:0/16:1; 14:0/18:1**	$[C_{35}H_{66}O_8P]^-$	**12.80**
3	667.4360	2.4	14:0/20:4	$[C_{37}H_{64}O_8P]^-$	4.18
4	671.4673	2.4	16:0/18:2; 18:1/16:1	$[C_{37}H_{68}O_8P]^-$	6.60
5	673.4825	1.6	16:0/18:1	$[C_{37}H_{70}O_8P]^-$	9.15
6	691.4354	1.4	18:3/18:3	$[C_{39}H_{64}O_8P]^-$	0.97
**7**	**695.4668**	**1.6**	**16:0/20:4; 18:2/18:2**	$[C_{39}H_{68}O_8P]^-$	**12.88**
8	719.4672	2.1	18:2/20:4	$[C_{41}H_{68}O_8P]^-$	2.63
9	741.4517	2.2	20:4/20:5	$[C_{43}H_{66}O_8P]^-$	0.33
**10**	**743.4671**	**1.9**	**20:4/20:4**	$[C_{43}H_{68}O_8P]^-$	**47.85**

Table S7. List of PE and LPE identified as deprotonated molecules, $[M-H]^-$, in the edible brown alga wakame by HILIC-ESI(-)-CID/MS/MS.The most abundant species are in bold.

#	Accurate *m/z*	Mass Error (ppm)	PE (*sn*-1/*sn*-2)	Molecular Formula [M-H]⁻	Relative Abundance (%)
1	660.4608	−0.3	18:1/12:0; 16:0/14:1; 14:0/16:1	$[C_{35}H_{67}NO_8P]^-$	1.29
2	662.4789	3.5	16:0/14:0	$[C_{35}H_{69}NO_8P]^-$	2.00
**3**	**686.4792**	**3.8**	**16:1/16:1**	$[C_{37}H_{69}NO_8P]^-$	**7.11**
**4**	**688.4929**	**0.9**	**16:0/16:1; 18:1/14:0**	$[C_{37}H_{71}NO_8P]^-$	**63.02**
5	710.4777	1.5	14:0/20:4; 16:0/18:4; 18:3/16:1	$[C_{39}H_{69}NO_8P]^-$	0.98
**6**	**714.5069**	**−1.4**	**18:1/16:1**	$[C_{39}H_{73}NO_8P]^-$	**9.68**
**7**	**738.5092**	**1.8**	**16:0/20:4**	$[C_{41}H_{73}NO_8P]^-$	**6.06**
8	742.5392	0.0	18:1/18:1	$[C_{41}H_{77}NO_8P]^-$	1.30
9	762.5080	0.1	18:2/20:4	$[C_{43}H_{73}NO_8P]^-$	0.90
10	764.5253	2.2	18:1/20:4	$[C_{43}H_{75}NO_8P]^-$	0.22
11	766.5388	−0.5	18:0/20:4	$[C_{43}H_{77}NO_8P]^-$	0.66
**12**	**786.5100**	**2.7**	**20:4/20:4**	$[C_{45}H_{73}NO_8P]^-$	**5.52**
13	788.5254	2.3	20:3/20:4	$[C_{45}H_{75}NO_8P]^-$	0.01
14	794.5687	−2.3	20:0/20:4	$[C_{45}H_{81}NO_8P]^-$	1.25
			**LPE**		
1	450.2638	2.7	16:1/0:0	$[C_{21}H_{41}NO_7P]^-$	11.45
2	452.2795	2.7	16:0/0:0	$[C_{21}H_{43}NO_7P]^-$	7.81
3	478.2950	2.3	18:1/0:0	$[C_{23}H_{45}NO_7P]^-$	2.12
4			0:0/18:1	$[C_{23}H_{45}NO_7P]^-$	0.26
5	480.3106	2.1	18:0/0:0	$[C_{23}H_{47}NO_7P]^-$	0.59
6	498.2645	3.8	20:5/0:0	$[C_{25}H_{41}NO_7P]^-$	5.23
7	**500.2797**	2.8	**20:4/0:0**	$[C_{25}H_{43}NO_7P]^-$	**65.07**
8			0:0/20:4	$[C_{25}H_{43}NO_7P]^-$	7.47

Table S8. List of PC and LPC identified in negative ion mode in the edible brown alga wakame by HILIC-ESI(-)-CID/MS/MS. The most abundant species are in bold.

#	Accurate *m/z*	Mass Error (ppm)	PC (*sn*-1/*sn*-2)	Adduct	Adduct Molecular Formula	Relative Abundance (%)
1	712.4918	−0.7	14:0/18:3	$[M-CH_3]^-$	$[C_{39}H_{71}NO_8P]^-$	0.21
2	714.5078	−0.1	14:0/18:2; 16:0/16:2; 16:1/16:1	$[M-CH_3]^-$	$[C_{39}H_{73}NO_8P]^-$	0.80
3	738.5075	−0.5	14:0/20:4; 16:0/18:4	$[M-CH_3]^-$	$[C_{41}H_{73}NO_8P]^-$	0.77
4	742.5389	−0.4	16:0/18:2	$[M-CH_3]^-$	$[C_{41}H_{77}NO_8P]^-$	1.76
5	766.5402	1.3	16:0/20:4; 18:2/18:2	$[M-CH_3]^-$	$[C_{43}H_{77}NO_8P]^-$	1.14
**6**	**774.5302**	**1.4**	**14:0/18:2; 16:0/16:2; 16:1/16:1; 19:2_13:0**	**$[M+HCOO]^-$**	**$[C_{41}H_{77}NO_{10}P]^-$**	**9.45**
7	786.5095	2.0	20:4/18:4	$[M-CH_3]^-$	$[C_{45}H_{73}NO_8P]^-$	0.10
8	788.5255	2.4	20:4/18:3; 20:5/18:2	$[M-CH_3]^-$	$[C_{45}H_{75}NO_8P]^-$	0.16
9	790.5406	1.8	20:4/18:2	$[M-CH_3]^-$	$[C_{45}H_{77}NO_8P]^-$	0.50
10	794.5698	−0.9	18:0/20:4	$[M-CH_3]^-$	$[C_{45}H_{81}NO_8P]^-$	0.07
**11**	**802.5614**	**1.2**	**16:0/18:2**	**$[M+HCOO]^-$**	**$[C_{43}H_{81}NO_{10}P]^-$**	**34.05**
12	814.5408	2.0	20:4/20:4	$[M-CH_3]^-$	$[C_{47}H_{77}NO_8P]^-$	0.45
13	822.6033	1.8	20:0/20:4	$[M-CH_3]^-$	$[C_{47}H_{85}NO_8P]^-$	0.02
**14**	**826.5598**	**−0.7**	**16:0/20:4; 18:2/18:2**	**$[M+HCOO]^-$**	**$[C_{45}H_{81}NO_{10}P]^-$**	**17.73**
15	848.5459	1.4	20:4/18:3; 20:5/18:2	$[M+HCOO]^-$	$[C_{47}H_{79}NO_{10}P]^-$	3.37
**16**	**850.5580**	**−2.8**	**20:4/18:2**	**$[M+HCOO]^-$**	**$[C_{47}H_{81}NO_{10}P]^-$**	**12.05**
17	852.5751	−1.1	20:4/18:1; 20:3/18:2; 20:5_18:0	$[M+HCOO]^-$	$[C_{47}H_{83}NO_{10}P]^-$	2.64
18	854.5908	−1.1	18:0/20:4; 18:1_20:3	$[M+HCOO]^-$	$[C_{47}H_{85}NO_{10}P]^-$	1.61
19	866.5916	−0.1	20:4/19:1; 21:3/18:2; 18:3_21:2	$[M+HCOO]^-$	$[C_{48}H_{85}NO_{10}P]^-$	0.03
20	872.5456	1.0	20:4_20:5	$[M+HCOO]^-$	$[C_{49}H_{79}NO_{10}P]^-$	2.81
21	874.5579	−2.9	20:4/20:4	$[M+HCOO]^-$	$[C_{49}H_{81}NO_{10}P]^-$	9.67
22	882.6234	0.5	20:0/20:4	$[M+HCOO]^-$	$[C_{49}H_{89}NO_{10}P]^-$	0.61
23	908.6352	−3.7	22:1/20:4	$[M+HCOO]^-$	$[C_{51}H_{91}NO_{10}P]^-$	0.19
			**LPC (*sn*-1/*sn*-2)**			
1	452.2795	2.7	14:0/0:0	$[M-CH_3]^-$	$[C_{21}H_{43}NO_7P]^-$	0.99
2	478.2938	−0.2	16:1/0:0	$[M-CH_3]^-$	$[C_{23}H_{45}NO_7P]^-$	0.56
3			0:0/16:1			0.07
4	480.3094	−0.4	16:0/0:0	$[M-CH_3]^-$	$[C_{23}H_{47}NO_7P]^-$	3.38
5			0:0/16:0			0.16
6	500.2793	2.0	18:4/0:0	$[M-CH_3]^-$	$[C_{25}H_{43}NO_7P]^-$	1.73
7	502.2955	3.2	18:3/0:0	$[M-CH_3]^-$	$[C_{25}H_{45}NO_7P]^-$	2.68
8			0:0/18:3			0.30
**9**	**504.3113**	**3.4**	**18:2/0:0**	**$[M-CH_3]^-$**	**$[C_{25}H_{47}NO_7P]^-$**	**11.17**
10			0:0/18:2			1.34
11	506.3260	1.6	18:1/0:0	$[M-CH_3]^-$	$[C_{25}H_{49}NO_7P]^-$	2.17
12			0:0/18:1			0.25
13	508.3403	−1.2	18:0/0:0	$[M-CH_3]^-$	$[C_{25}H_{51}NO_7P]^-$	0.30
14			0:0/18:0			0.01
15	526.2954	2.9	20:5/0:0	$[M-CH_3]^-$	$[C_{27}H_{45}NO_7P]^-$	1.49
16			0:0/20:5			0.18
17	528.3095	−0.2	20:4/0:0	$[M-CH_3]^-$	$[C_{27}H_{47}NO_7P]^-$	8.71
18			0:0/20:4			0.96
19	530.3264	2.3	20:3/0:0	$[M-CH_3]^-$	$[C_{27}H_{49}NO_7P]^-$	1.07

20			0:0/20:3			0.14
21	532.3421	2.3	20:2/0:0	$[M\text{-}CH_3]^-$	$[C_{27}H_{51}NO_7P]^-$	0.19
22	540.3320	2.4	16:0/0:0	$[M+HCOO]^-$	$[C_{25}H_{51}NO_9P]^-$	5.37
23	562.3165	2.7	18:3_0:0	$[M+HCOO]^-$	$[C_{27}H_{49}NO_9P]^-$	5.41
**24**	**564.3323**	**2.8**	**18:2/0:0**	**$[M+HCOO]^-$**	**$[C_{27}H_{51}NO_9P]^-$**	**23.71**
25	566.3464	0.2	18:1_0:0	$[M+HCOO]^-$	$[C_{27}H_{53}NO_9P]^-$	4.70
26	568.3616	−0.7	18:0_0:0	$[M+HCOO]^-$	$[C_{27}H_{55}NO_9P]^-$	0.61
**27**	**588.3323**	**2.7**	**20:4/0:0**	**$[M+HCOO]^-$**	**$[C_{29}H_{51}NO_9P]^-$**	**22.32**

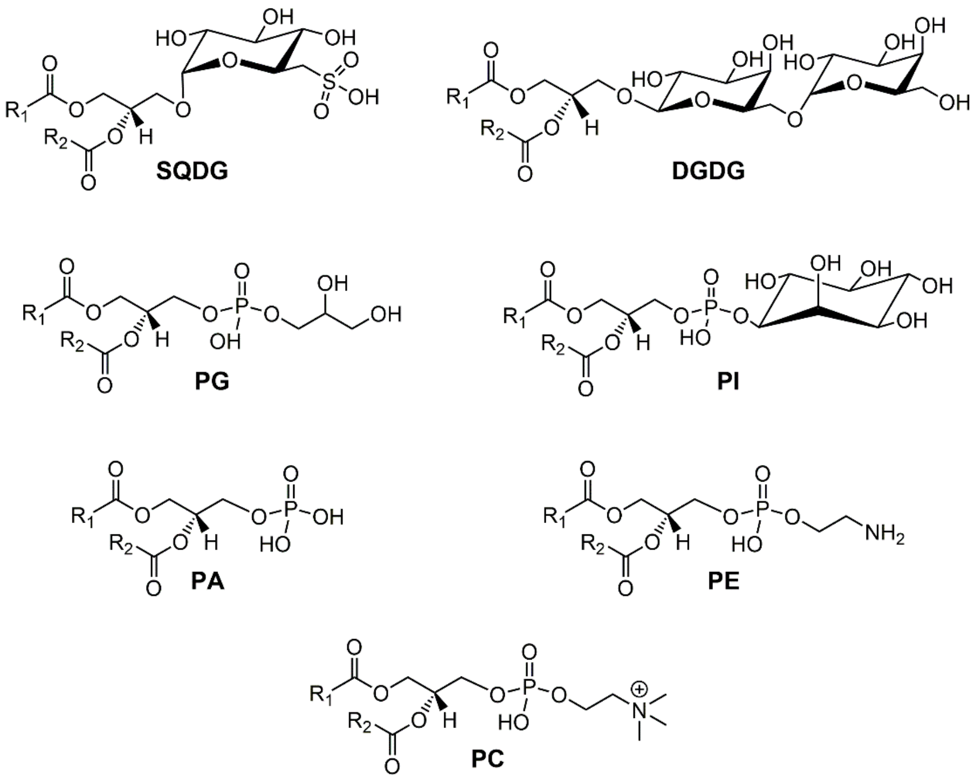

Figure S1. Molecular structures of the investigated lipid classes. Sulfoquinovosyl diacylglycerols (SQDG), digalactosyldiacylglycerols (DGDG), phosphatidylglycerols (PG), phosphatidylinositols (PI), phosphatidic acids (PA), phosphatidylethanolamines (PE), and phosphatidylcholines (PC).

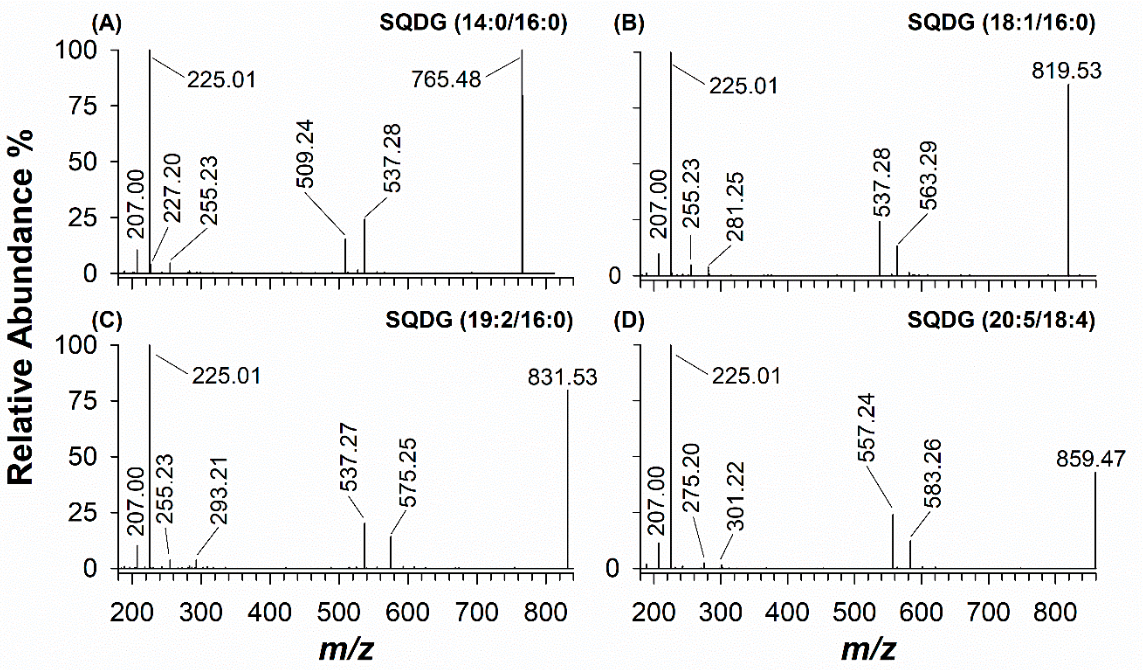

Figure S2. RPLC-ESI(-)-FTMS/MS spectra of the ions at (**A**) *m/z* 765.48, SQDG 14:0/16:0, (**B**) *m/z* 819.53, SQDG 18:01/16:0, (**C**) *m/z* 831.53, SQDG 19:2/16:0 and (**D**) *m/z* 859.47, SQDG 20:5/18:4.

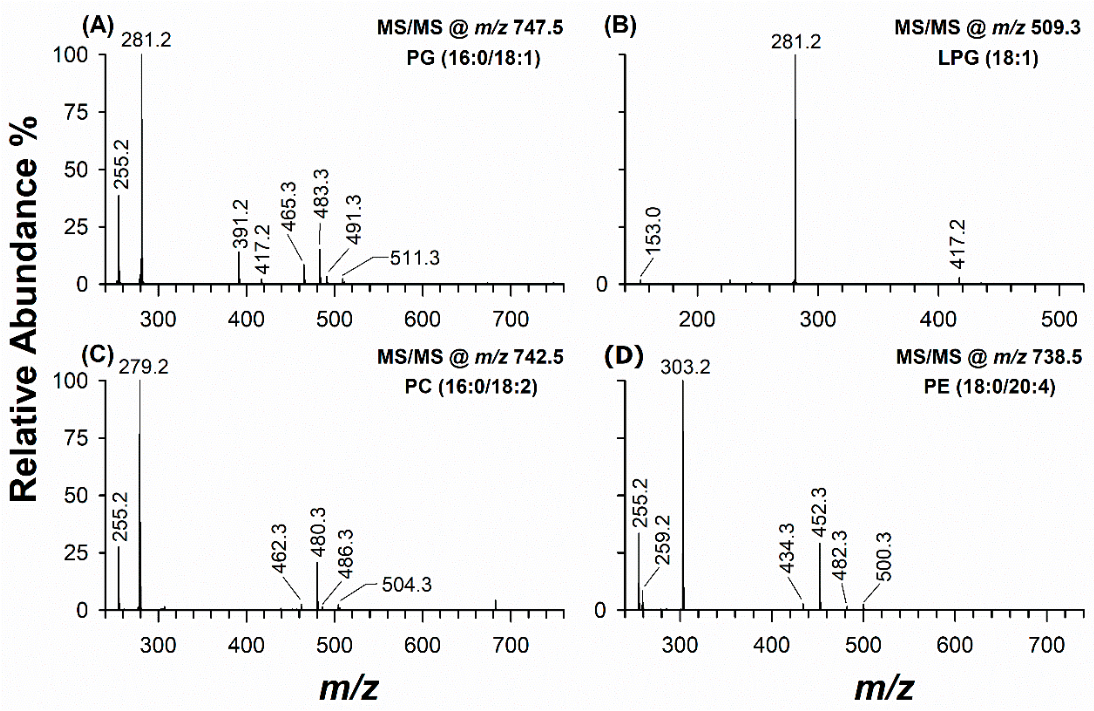

Figure S3. HILIC-ESI(-)-FTMS/MS spectra of the ions at (**A**) *m*/*z* 747.5, PG 16:0/18:1, (**B**) *m*/*z* 509.3, LPG 18:1, (**C**) *m*/*z* 742.5, PC 16:0/18:2, and (**D**) *m*/*z* 738.5, PE 18:0/20:4.

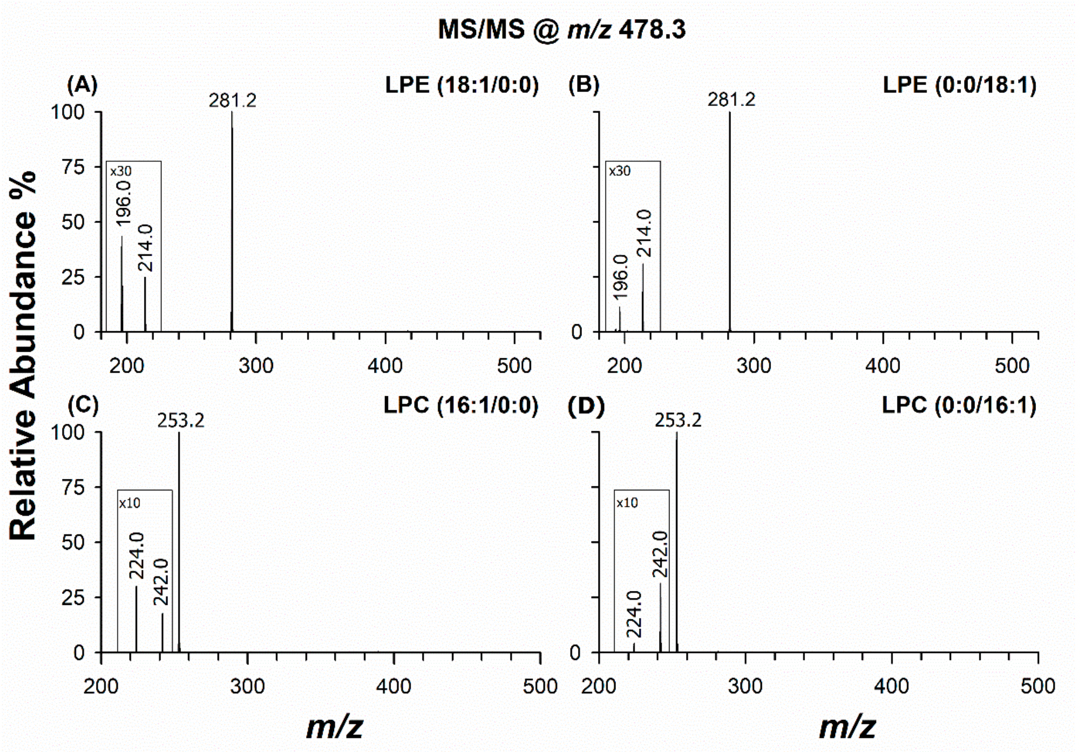

Figure S4. HILIC-ESI(-)-ITMS/MS spectra averaged under the four chromatographic peaks related to the ion at *m*/*z* 478.3, identified as (**A**) LPE 18:1/0:0, (**B**) LPE 0:0/18:1, (**C**) LPC 16:1/0:0, and (**D**) LPC 0:0/16:1.
